# Supplementary material for: Vibrio cholerae serotype impacts pathogenicity
Source: Nat Commun. 2026 Jan 19;17:1140. doi: 10.1038/s41467-025-67908-w (PMC12856009; doi:10.1038/s41467-025-67908-w)
Supplement: Supplementary file 1 — Supplementary Information [file 41467_2025_67908_MOESM1_ESM.pdf]

Supplementary information

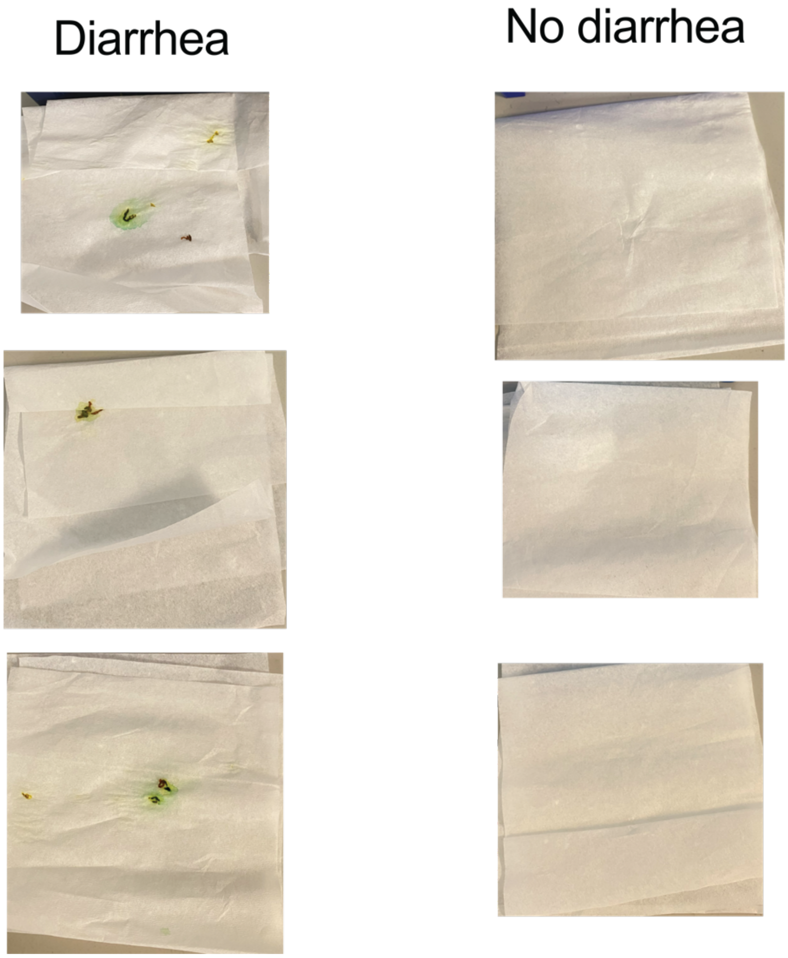

Supplementary Figure 1: **Indications of diarrhea.** Representative images of suckling mouse beddings identified with or without diarrhea. All mice used were Crl:CD1(ICR) mixed sex, postnatal day 5 at the time of infection.

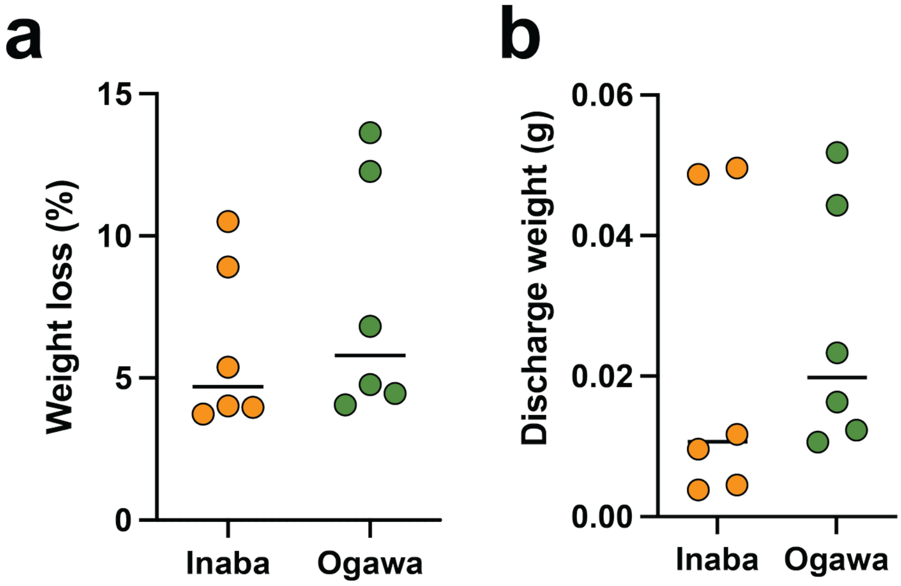

Supplementary Figure 2: **Measurements of weight loss and diarrheal discharge.** Weight loss (c) and weight of diarrheal discharge (d) of p5 suckling mice after 24 of infection with Inaba (orange) or Ogawa (green) serotype. All mice used were Crl:CD1(ICR) mixed sex, postnatal day 5 at the time of infection (n = 6). Source data are provided as a Source Data file.

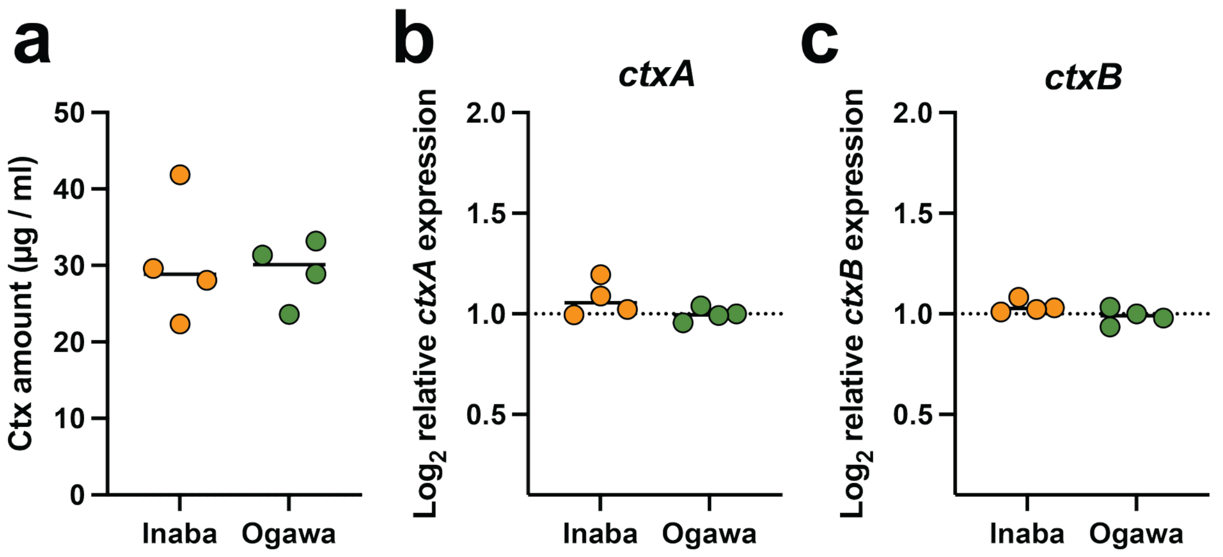

Supplementary Figure 3: **Impact of serotype on Virulence expression**. (a) amount of cholera toxin was measured by CTX ELISA of the Inaba (orange) or Ogawa (green) serotype grown in AKI medium (n = 4). qRT-PCR measurements of ctxA (b) and ctxB (c) of the Inaba (orange) or Ogawa (green) serotype after 18 h of infection of suckling mice (n = 4). All mice used were Crl:CD1(ICR) mixed sex, postnatal day 5 at the time of infection. Source data are provided as a Source Data file.

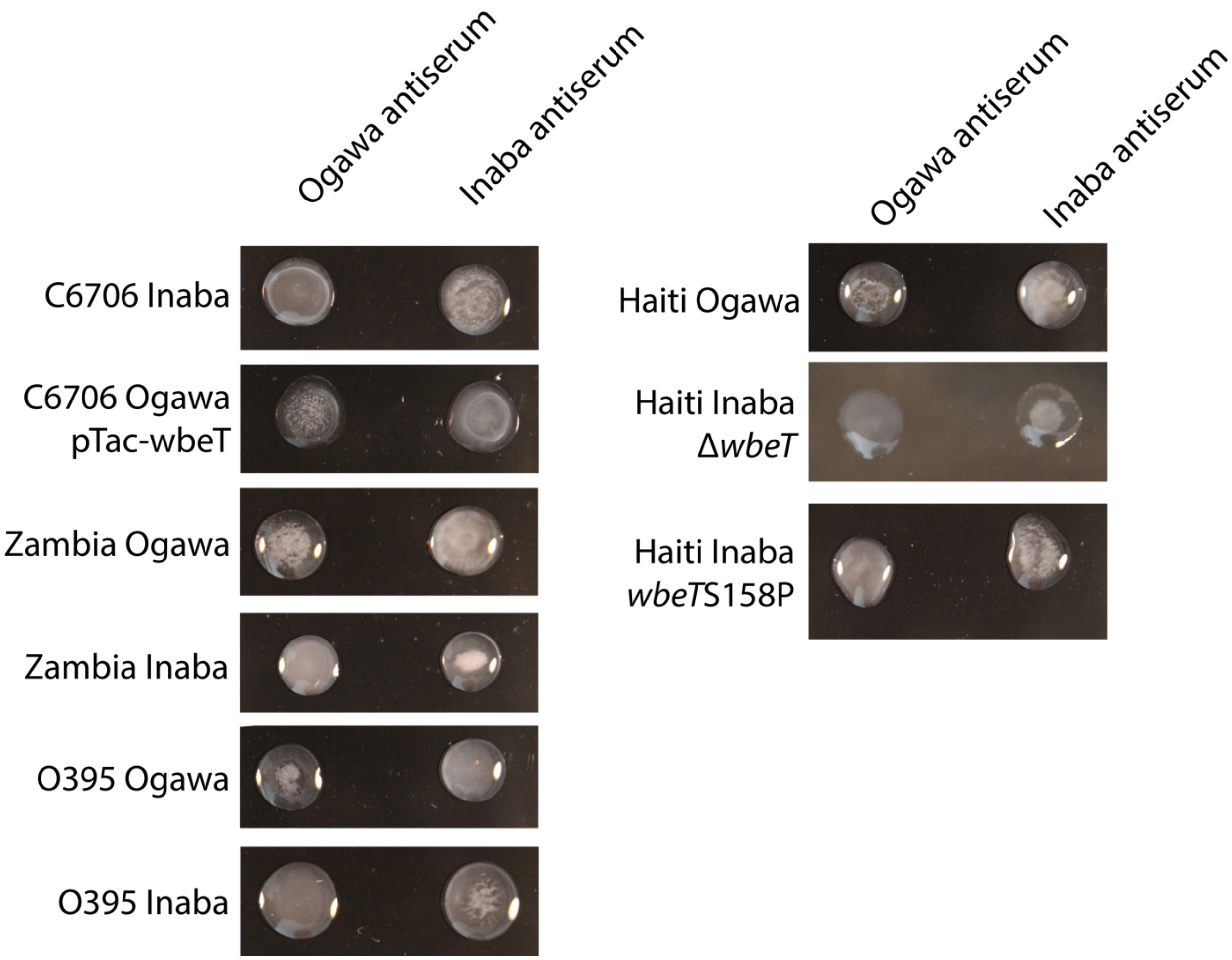

Supplementary Figure 4: **Serotyping of *V. cholerae* isolates used in this study**. *V. cholerae* cultures treated with Ogawa or Inaba specific antisera. Images were taken after 5-10 minutes of incubation at room temperature.

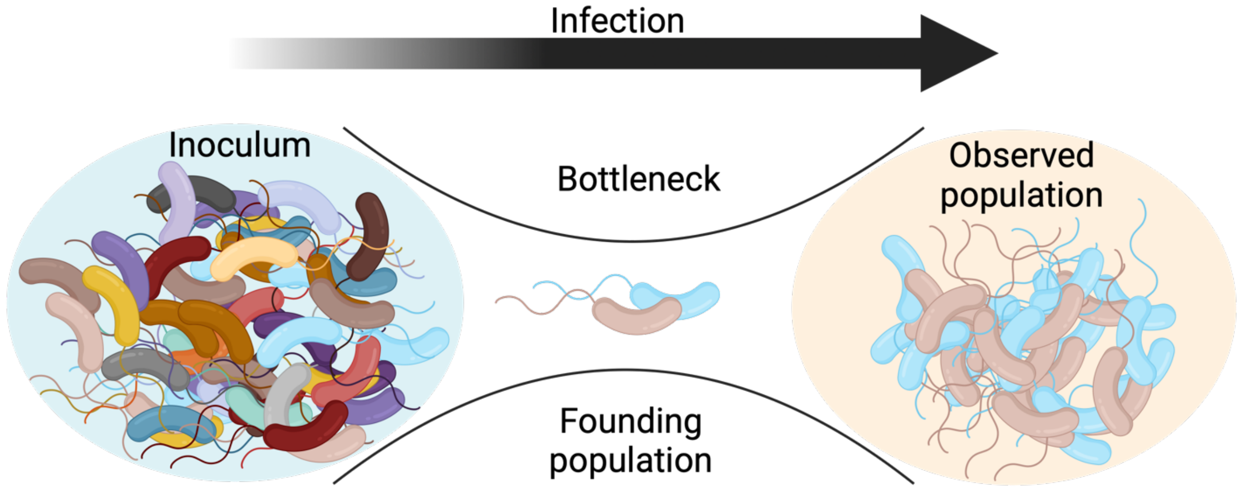

Supplementary Figure 5: **Schematic depicting assays to measure founding populations.** Barcoded bacteria are used to measure the number of bacteria that survive the bottleneck, the founders, and give rise to the population in intestinal homogenates. Parts of this figure were created in BioRender. Zingl, F. (2025) https:// BioRender.com/fhbbff0.

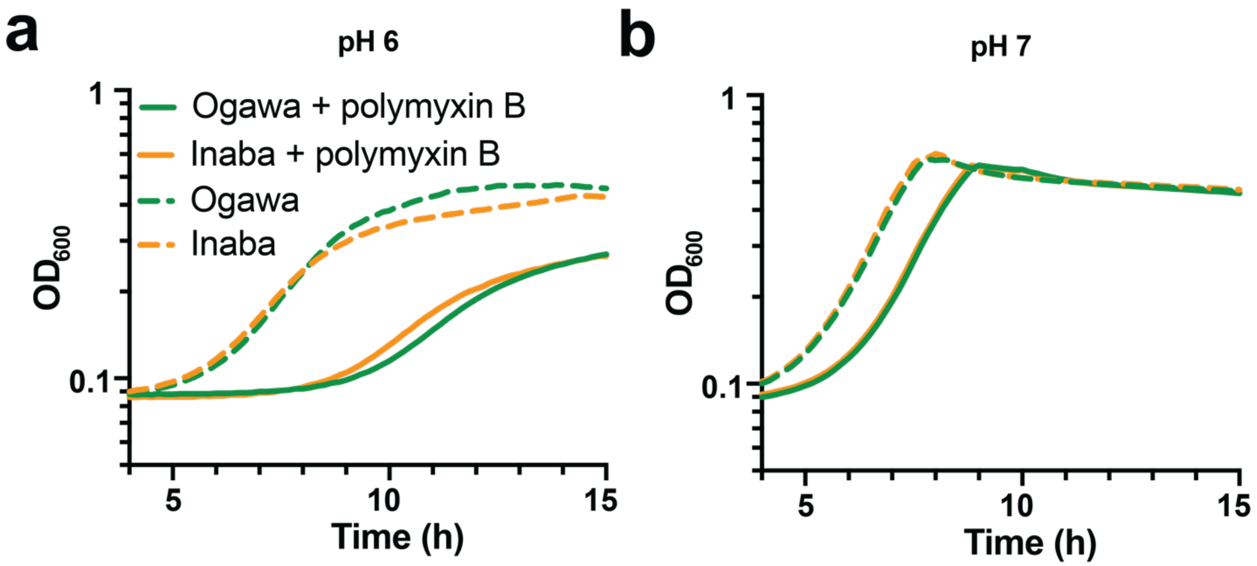

Supplementary Figure 6: **Growth curves at pH 6 and pH 7.** Growth curves at pH 6 (a; n = 3) and pH 7 (b; n = 4) of Inaba (orange) or Ogawa (green) strains in M9 with (solid line) or without (dashed line) polymyxin B. Source data are provided as a Source Data file.

Supplementary Table 1: Bacterial strains used in this study.

Strain	Genotype	Source or reference
E.coli		
MFDλpir	pSM1; CbR, Km^R	27
MFDλpir	pJMP1039; Cb^R	27
Sm10λpir	pCVD442::Δ*wbeT* ; Cb^R	27
Sm10λpir	pCVD442::Δ*lacZ* ; Cb^R	28
Sm10λpir	pCVD442::pTac *wbeT* ; Cb^R	This study
Sm10λpir	pCVD442::*wbeT* S158P; Cb^R	28
V. cholerae		
Haiti 2010 Ogawa	wild-type *V. cholerae* strain serogroup O1; serotype Ogawa; spontaneous Sm^R mutant of a clinical isolate of Haiti 2010	Lab collection
Haiti 2010 Ogawa Δ*lacZ*	Haiti 2010 Ogawa Δ*lacZ* ; Sm^R	28
Haiti 2010 Inaba (*wbeT*- S158P)	Haiti 2010 Ogawa *wbeT*- S158P; Sm^R	This study
Haiti 2010 Inaba Δ*wbeT*	Haiti 2010 Ogawa Δ*wbeT* ; Sm^R	This study
C6706 Inaba	wild-type *V. cholerae* strain serogroup O1; serotype Inaba; Peru 1991; Sm^R	Lab collection
C6706 Inaba Δ*lacZ*	C6706 1991 Peru Inaba Δ*lacZ* ; Sm^R	This study
C6706 Ogawa (pTac wbeT)	C6706 1991 Peru pTac *wbeT* in STAMP locus; Sm^R	This study
Zambia 2016 Ogawa	wild-type *V. cholerae* strain serogroup O1; serotype Ogawa; Zambia 2016; Sm^R	27
Zambia 2016 Inaba Δ*wbeT*	ZambiaWT 2016 Ogawa Δ*wbeT* ; Sm^R	27
Zambia 2016 Inaba Δ*wbeT* Δ*lacZ*	ZambiaWT 2016 Ogawa Δ*wbeT Δ lacZ* ; Sm^R	This study
O395 Ogawa	wild-type *V. cholerae* strain serogroup O1; classical; serotype Ogawa; Sm^R	Lab collection
O395 Ogawa Δ*lacZ*	Classical Ogawa Δ*lacZ* ; Sm^R	This study
O395 Inaba	Classical Ogawa Δ*wbeT* ; Sm^R	This study
